# Residue correlation networks in nuclear receptors reflect functional specialization and the formation of the nematode-specific P-box

**DOI:** 10.1186/1471-2164-14-S6-S1

**Published:** 2013-10-25

**Authors:** Marcelo Querino Lima Afonso, Leonardo Henrique França de Lima, Lucas Bleicher

**Affiliations:** 1 Universidade Federal de Minas Gerais, Belo Horizonte, Brazil; 2 Universidade Federal de São João del-Rei, Sete Lagoas, Brazil

## Abstract

**Background:**

Nuclear receptors (NRs) are transcription factors which bind small hormones, whose evolutionary history and the presence of different functional surfaces makes them an interesting target for a correlation based analysis.

**Results:**

Correlation analysis of ligand binding domains shows that correlated residue subsets arise from the differences between functional sites in different nuclear receptor subfamilies. For the DNA binding domain, particularly, the analysis shows that the main source of correlation comes from residues that regulate hormone response element specificity, and one of the conserved residue sub-sets arises due to the presence of an unusual sequence for the DNA binding motif known as P-box in nematodes, suggesting the existence of different DBD-DNA specificities in nuclear receptors.

**Conclusions:**

We conclude that DNA specificity and functional surface specialization has independently driven nuclear receptor evolution, and suggest possible binding modes for the class of divergent nuclear receptors in nematodes.

## Background

The large family of nuclear receptors (NRs) comprise regulatory transcription factors that are activated by specific ligands (usually small lipophilic molecules) and regulate a wide range of biological processes in metazoa, and the association of many of them to human diseases make them a current major drug target[1]. The overall architecture of NRs consists of an N-terminal region (A/B domain), a DNA binding domain (DBD, or C domain), a molecular hinge (D domain), a ligand binding domain (LBD) and a C-terminal region (F domain). Given the functional importance of the LBD (where hormone binding is responsible for the structural changes and recruitment of other proteins for transcription initiation) and the DBD (which selectively binds to the DNA sequence known as the hormone response element, or HRE), they are the most conserved domains and are easily alignable (the DBD being the most conserved region). On the other hand, all other domains are highly variable in size and sequence (being even absent in some cases). There are also NRs lacking LBDs (as Knirps) or DBDs (as DAX-1, which has regulatory function by heterodimerization). More recently, nuclear receptors containing two DBDs have also been identified from the flatworm parasite *Schistosoma mansoni *and database mining have detected this architecture not only in other platyhelminthes but also in mollusks and arthropods [2]. Such studies are possible due to the increasingly high availability of NR sequences - the current PFAM [3] release lists 4842 DBDs and 4622 LBDs from and 511 and 481 species, respectively. For both domains, more than 90% of the available sequences come from nematoda, chordata and arthropoda.

Phylogenetic analysis divide nuclear receptors in six classes, named NR1-NR6, plus a seventh class (NR0) for the unusual nuclear receptors lacking either the DBD or LBD. Nuclear receptors may also be referred to as Type I-IV: Type I receptors, comprising the NR3 subfamily, are cytosolic and ligand binding causes its transport to the nucleus after dissociation from heat shock proteins and homodimerization. Type II receptors, which correspond to the NR1 class, are kept in the nucleus, usually binding to DNA as heterodimers (RXR is the usual heterodimerization partner). They are inactivated by co-repressors in the absence of ligands, which, when present, cause the dissociation from co-repressors and co-activator recruiting. Type III receptors (NR2) are similar to Type I, the difference relying on the HRE sequence (Type I receptors bind to inversed repeats, while Type III bind to direct repeats). Finally, Type IV receptors are able to bind to DNA as monomers also, and are not restricted to a single class in the NR1-NR6 nomenclature.

The *Caenorhabditis elegans *(*C.elegans*) nematode is a particularly interesting model organism for the study of nuclear receptor evolution, due to the fact that its number of predicted NRs is above 300, 75% of them possibly representing functional genes [4]. This is the highest number of NR genes ever found in a single species to date, being considerably higher than the amount found in *Drosophila *(~20) and even in humans (~50). The *Caenorhabditis briggsae *species, which also has available sequences, presents such a high amount of potential nuclear receptor genes as well. Although abundant, most *C. elegans *NR genes are difficult to be grouped in the NR1-NR6 system, which characterize a distinct event of proliferation and diversification for those receptors, probably from a series of duplications of a HNF4 ancestor, an orphan receptor [5]. From all known *C. elegans *nuclear receptors, only seven have well described functions [4].

Protein families can be usually identified by well conserved motifs. In the case of nuclear receptors, DBDs can be easily detected by the presence of two highly conserved C4 zinc finger motifs, while LBDs contain a twenty residue long "signature motif" that stabilizes its canonical fold [6]. Aside from positional conservation, which can define such motifs or detect family-wide functional important residues (such as those involved in catalysis in the case of enzymes), another useful way of studying protein families is looking for correlations - i.e., the fact that a given residue in a certain position in a multiple sequence alignment (MSA) increases or decreases the chance of observing another residue in another position. Quantitatively measuring such correlations was made possible due to the availability of a sufficient number of sequences from a given protein family, and many studies based on correlated mutations have appeared, especially during the nineties, following the seminal article of Göbel and co-authors in 1994 [7]. This was followed by other correlation metrics such as mutual information[8], statistical coupling analysis[9], explicit likelihood [10], etc. Statistical coupling analysis have been applied to the study of LBDs [11], while the alignable portions of full-length NRs were studied by mutual information [12], which resulted in successfully reporting residues that connect the functionally important surfaces in nuclear receptors [11] and a set of three residues in the dimer interface that uniquely identify nuclear receptors [12]. We have previously observed that further analysis of correlated positions in a multiple sequence analysis may reveal class-determining patterns - specifically, residues involved in metal selectivity and oligomeric state in Fe/Mn-Superoxide Dismutases [13], while Halabi *et al. *independently detected the existence of "sectors" in protein family multiple sequence analysis that may also evolve independently and be responsible for different functions [14]. A new methodology was proposed in order to extract this additionally available and potentially useful information from correlation analysis [15] - specifically, we adopted the independent calculation of residue-specific correlation (which also enables calculation of the usually overlooked cases of anti-correlation), so that different class-determining groups are not masked by their potential presence in the same position in a multiple sequence alignment, which would be a limitation for metrics that report interpositional correlation (possibly followed by a clustering procedure) only. A correlation network where nodes are residue-position pairs (as in D48, E93 or K93) and connections are (anti-)correlation scores (e.g. a positive link between D48 and K93 means the presence of one of those residues significantly increases the odds of finding the other, while a negative link means they are unlikely to be simultaneously present in a given protein) is built and subsequently decomposed (using, for example, techniques from community structure analysis [16-18]) in order to detect residue groups (with their type explicitly reported) that may be related to a class-specific function or characteristic [15]. The presence of well-defined functional surfaces, very different modes of action and a remarkable evolutionary history [19] led us to believe that correlation network decomposition could provide useful residue-specific information about the evolution and function of nuclear receptors.

## Results and discussion

### Ligand binding domains

Although nuclear receptor ligand binding domains have already been studied using correlation analysis [11], new insights can still be found when residue-specific metrics are used. The calculation and subsequent decomposition of the overall correlation network in LBDs resulted in four communities (a *community *in a network is a set of nodes which are well connected between themselves but not to the rest of the network; in a residue correlation network it consists of a set of residues which tend to appear simultaneously in a sub-set of a protein family). The first one consists of positions Ala283, Glu307, Phe289, Leu301, Pro287 and Gln297 (hRXRα numbering). Glu307 is close to the hormone binding site and all human nuclear receptors have an acidic residue in that position except for the steroid receptors and the two NRs lacking a DBD, DAX1 and SHP. The other residues in this community are close to the co-activator binding interface and also present in most human nuclear receptors.

The same is not true for the second community, consisting of residues Phe353, Gly288, Arg312 and Arg380 - whose individual frequencies are among 22-36% among all receptors. With residues (Gly288, Arg380) that are in contact with those in the co-activator binding site and also other two which are closer to the hormone binding site (Arg312, Phe353), they are not common to most human nuclear receptors, but actually present in specific NRs, possibly due to being involved in ligand specificity: in RAR, for example, a mutation in Arg272 (equivalent to position 312 in hRXRα numbering) affects ligand binding and causes resistance to all-trans retinoic acid [20], while in VDR the equivalent Arg274 is related to vitamin D binding, which turns undetectable upon its mutation to alanine [21]. Another class-specific characteristic related to residues in this second community is found for Arg380: it is involved in a salt bridge which is specific for Class II nuclear receptors [22] - in the case of RARα, for example, the equivalent Arg339 binds to Asp267. In this receptor, residue Phe312 (position 353 in RXRα), is involved in retinoic acid expulsion [23].

The third community presents residues Lys371, Leu276, Trp282 and Trp305, which are present in about one half of all receptors in our final alignment. Positive residues in position 371 are found in a buried salt bridge that is present in some NRs (in RXRα, the charged pair is Arg371 and Glu239), while a leucine in position 354 (equivalent for 276 in RXRα) may be part of the binding site to the metastasis tumor-associated 1estrogen receptor repressor [24]. *In silico *studies [25] suggested that the two tryptophans may be an important part of the estrogen receptor region that could bind polyproline-II containing proteins (which could result in the activation of mitogen activated protein kinases). These two tryptophans are extremely high correlated: the presence of a tryptophan in position 305 raises the frequency of tryptophans in position 282 from 50.5% to 84.5% (p < 10^-40^), in agreement with a possible important role for such residues as suggested by Jacquot *et al. *[25].

Finally there is a two-residue community consisting of Lys302 and Leu369, present individually in about 40% of the NR sequences. The role of these two residues has been well characterized for VDR. Using VDR numbering, Lys264 is crucial for ligand-dependent transactivation [26], while the mutation of Leu332 severely impairs its function, with all functions (ligand binding, heterodimerisation and gene transactivation ) abolished when Leu325 is also mutated [27].

### DNA binding domains

**Figure 1:**
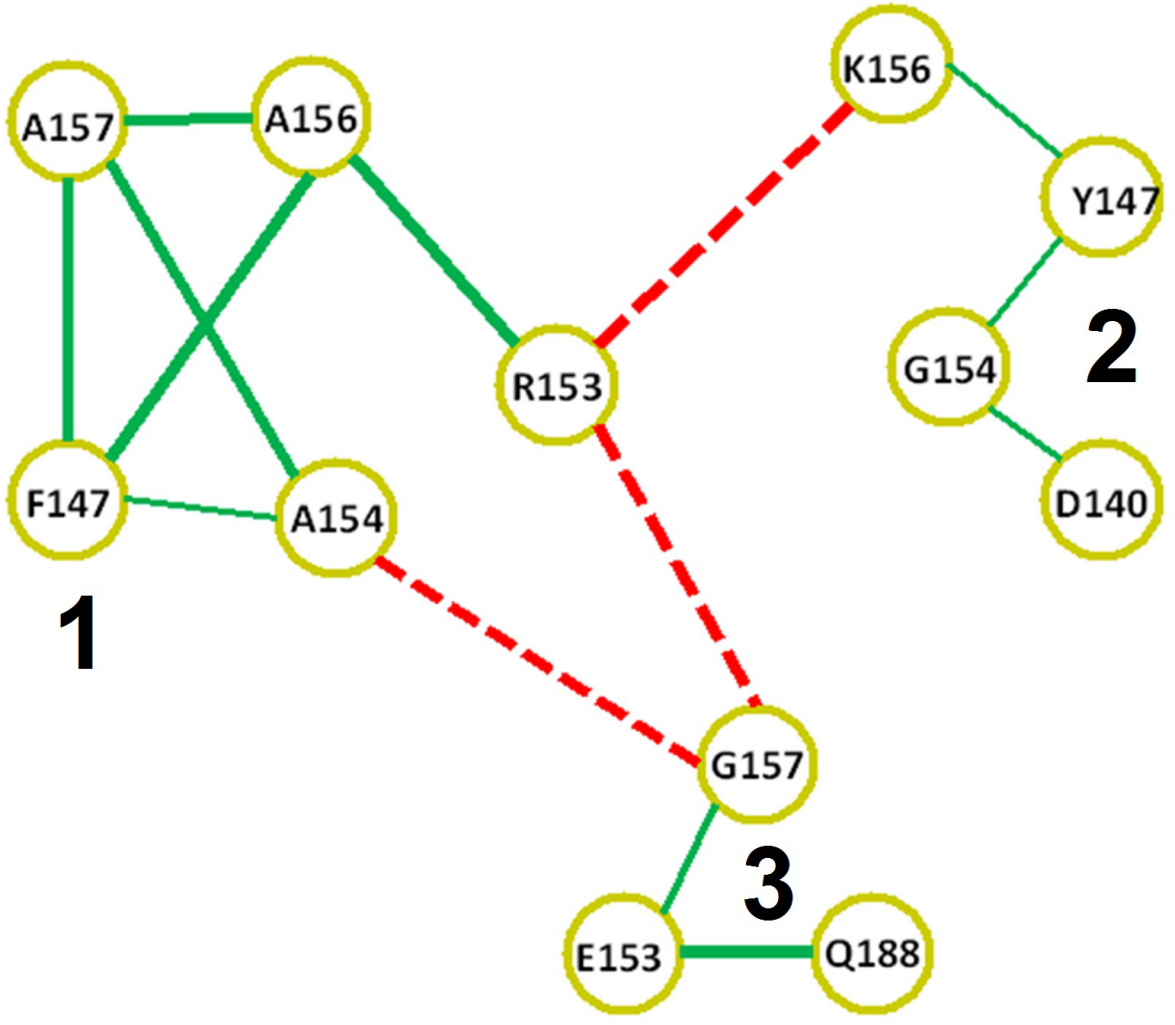
**Correlation network for nuclear receptor DBDs. Nodes are residue-position pairs using hRXRα numbering**. Connection widths are proportional to the correlation score. Positive scores are shown as green lines, negative scores (anti-correlation) are shown as dashed red lines.

**Figure 2:**
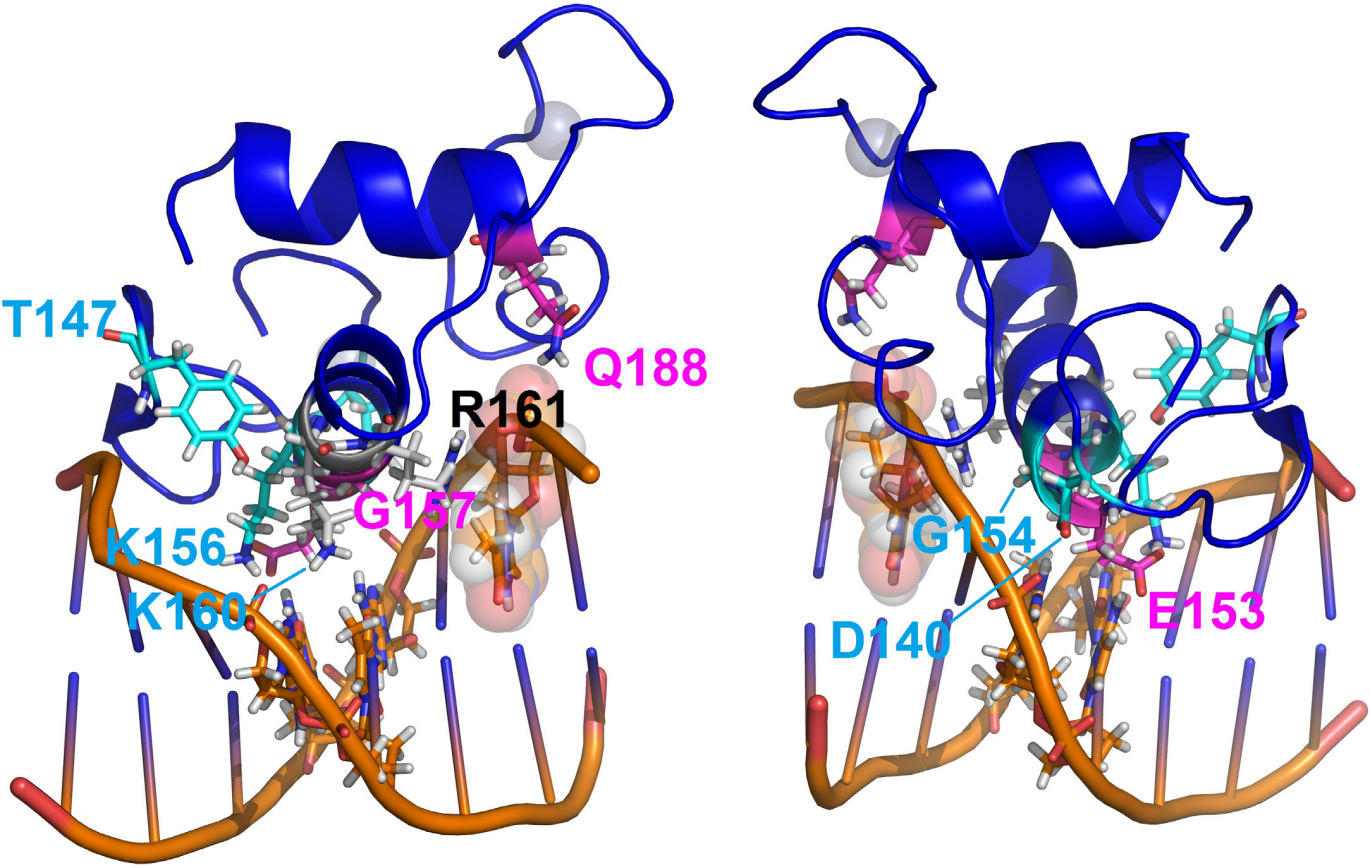
**Two side views of a representative structure of a canonical DBD:Half site complex presenting the residues from set 2 (carbon atoms in cyan blue), set 3 (in purple) and the conserved basic residues K160 and R161 (in white; letters in black)**. This color pattern will be used for all the figures along the paper, unless noted). The structure used was extracted from PDB 2NLL, showing RXRα-DBD complexed to a 5'-AGGTCA-3' half-site.

The correlation pattern among DBD residues is shown in Figure 1. Three sets are distinctly recognized from the figure: the first containing residues F147, R153, A154, A156 and A157, the second containing residues D140, Y147, G154, K156, and a third containing residues E153, G157, Q188 (hRXRα numbering used throughout the text unless noted). It is also visible from the network that subset 1 antagonizes the other two. Therefore, proteins containing the residues in this subset usually will not present the residues listed in the other two, in most cases due simply to different residue options in the same position (i.e., the presence of Phe147, Arg153, Ala154, Ala156 and Ala157 automatically means Tyr147, Glu153, Gly154, Lys156 and Gly157 are not allowed), but also by an apparent transitivity for Asp140 and Gln188. The 153-158 stretch can be directly mapped to a very important functional motif in DNA binding domains: the *P-box*, at the C-terminal region of the recognition helix (H-A), which directly interacts with DNA and is responsible for *half-site *specificity (Figure 2). P-boxes in human nuclear receptors usually have a CE^153^G^154^CK^156^G^157 ^or similar sequence (hRXRα numbering), except for the 3-ketosteroid class receptors (glucocorticoids, mineralocorticoids, progesterone and androgen receptors), which present a CG^153^S^154^CK^156^V^157 ^(or similar) sequence. The two cysteines in those sequences are strictly conserved in all DBDs, since they are necessary to bind zinc in the first of the two zinc fingers in this domain. Positions 2, 3 and 6 in the motifs shown above (respectively, 153, 154 and 157 on hRXRα) are called the 1^st^, 2^nd ^and 3^rd ^P-box positions, and site directed mutagenesis has shown that these three positions act concertedly to discriminate between half-sites on the HREs that differ in sequence at the third and fourth base pairs (AGNNCA, *N *being any nucleotide), such that substitutions at one position may alter the functionality of the others [28-30]. One of such studies, using the β isoform of the human thyroid receptor (hTRβ), has demonstrated that, in order to bind with high affinity to everted repeat sequences containing the AGGNCA hormone response element motif (TRevPal), a glutamate is required in the first P-box position, and also a glycine or alanine in the second one [29]. Beyond this, the topological context of the HRE neighborhood seems to influence the recognition mediated by these three positions. For example, in a similar study with hTRβ, the nature of the flanking sequence between the two half-sites (that does not make any direct contact with the P-box) showed itself influence on the lability of 1^st^, 2^nd ^and 3^rd ^P-box positions (with the more dramatic effects for the first one and only subtle effects for the 3^rd ^position) at the recognition of AGGNCA half-sites [30].

The residues in group 3 also includes Gln188, which lies on another motif known as Distal Box or simply D-box, a region in the second zinc finger which is perpendicular to the P-box and mediates dimerization. Community 2 includes also Tyr147, at the β-hairpin on the vicinity of the first zinc finger and making important contacts with the major groove backbone; and D140, at the first zinc finger, facing the DNA backbone at the 3' side, as Q188 (but, in opposition to this same residue, at a significant distance of 5-7 Å of the cited backbone). Our structural analysis points to an apparent more controversial role for this residue along the DBD evolution - the apparent nature of the correlations found at the 3 sets of the DBD correlated analysis are detailed at the next topics.

#### The DBD's correlation network reflects long range stereochemical adjustments on HRE recognition

**Figure 3:**
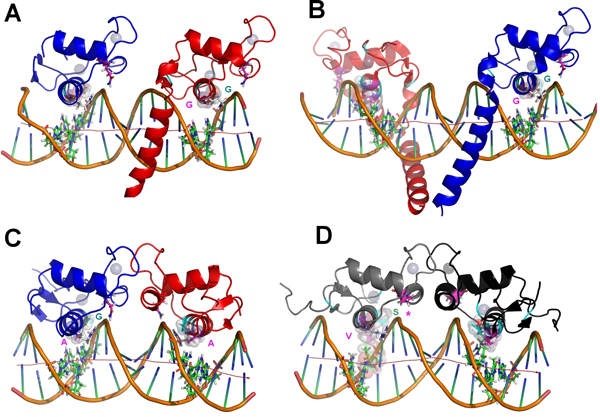
**Divergence on position 154 from set 2 and position 157 from set 3 are correlated with distortions on the HRE global topology**. *A *and *B *are, respectively the hRXRα/hTRβ:DR4 heterodimer and the hTRβ homodimer in an everted palindrome, both presenting short chain glycine residues at positions 154 and 157 (volumes shown as van der Walls transparent spheres) and an straight axis at the DNA topology. In *C*, the bulkier alanine side chain at position 157 from hER is well fitted to the ERE bent axis. In *D*, the higher distortion on the GRE axis (due the central AA kink) is accompanied by the bulkier G154S and G157V substitutions, and also a reduction on the chain at the 188 position with a Q-P exchange (asterisk). It can be noted the packing of the bulky V157 against the fourth anti-sense thimidine at the axis kink.

In order to better understand how the set of correlations recovered here can reflect on DBD functionality, we have analyzed representative structures of DBD:DNA complexes from literature, with "canonical" and divergent patterns of HRE recognition. In Figure 3, a representative structure of the hRXRα-DBD:HRE complex (from PDB 2NLL) is depicted, and residues contained in the sets 2 and 3 from the correlation network are shown, among two strongly conserved basic residues at the H-A's N-terminal: K160 (that is less frequently replaced by an R) and R161 (virtually ubiquitous among all members of the NR superfamily). The above cited set of residues and their accompanying protein:DNA contacts (when present) comprise the most conserved pattern at these respective positions between the non-3-ketosteroid receptors (the basic residues at the positions 160-161 being also extensive to the 3-ketosteroid receptors and other divergent NR classes - see below). The set 1 of our correlation analysis, on the other hand, is restricted to a very particular phylogenetic clade, in the nematode *Caenorhabditis *genus, whose apparent effects of its individual components will be gradually discussed later as they appear at some structures analyzed here, and then eventually considered as a whole group.

Firstly, considering just the 1^st^, 2^nd ^and 3^rd ^"canonical" P-box positions at Figure 3 (E153 from community 3; G154 from community 2 and G157, also from community 3), it can be noted that just the first one between them make apparent significant and specific hydrogen bonds with the nitrogenous basis at the third basis-pair and have its aliphatic chain in proximity at the fourth basis-pair. Such apparent discrepancy is taken off, however, when we consider the adjustments of glycines 154 and 157 at the HRE major groove on a topological context. G154 is in close proximity of the DNA backbone wall at the 5' extremity of the DNA anti-sense strand (sequence 5'-TGACCT-3') and near, but in the opposite face, of the sites where the E153 side chain contacts the nitrogenous basis on the major groove (Figure 3). In turn, G157 is closely surrounded by the aliphatic side chains of the basic residues K156 (from set 2 of the correlation network), K160 and R161. From these three, R161 is strongly packed over the C7 atom of the final anti-sense thymidine and interacts with the phosphate-backbone at the great majority of the NR:HRE structures; K156 makes a direct saline bond with the already mentioned E153 side chain (in agreement with the strong anti-correlation between K156 and an E153R substitution, as seen in Figure 1) and hydrogen bonds with the second basis pair at the half-site; while K160 makes moderate interactions with nucleotides at the two central basis-pairs. Such highly packed system combined with the relatively limited space of the major groove causes stereochemical restrictions over the side chains that can be accommodated at the positions 153 and 157 in order to maintain the system interactions on their total integrity. On the study of Nelson *et al. *[29], the nature of the substitutions at these positions that were permissive for the recognition of a set of AGNNCA variants were congruent with stereochemical clash at these sites. The apparent concerted way in which the two P-box glycine residues adapt themselves to the stereochemical ambient at the DBD-major groove complex is also suggested by the anti-correlation between G157 (at set 3) and the G154A substitution (at set 1), as recovered from our correlation network (Figure 1).

**Figure 4:**
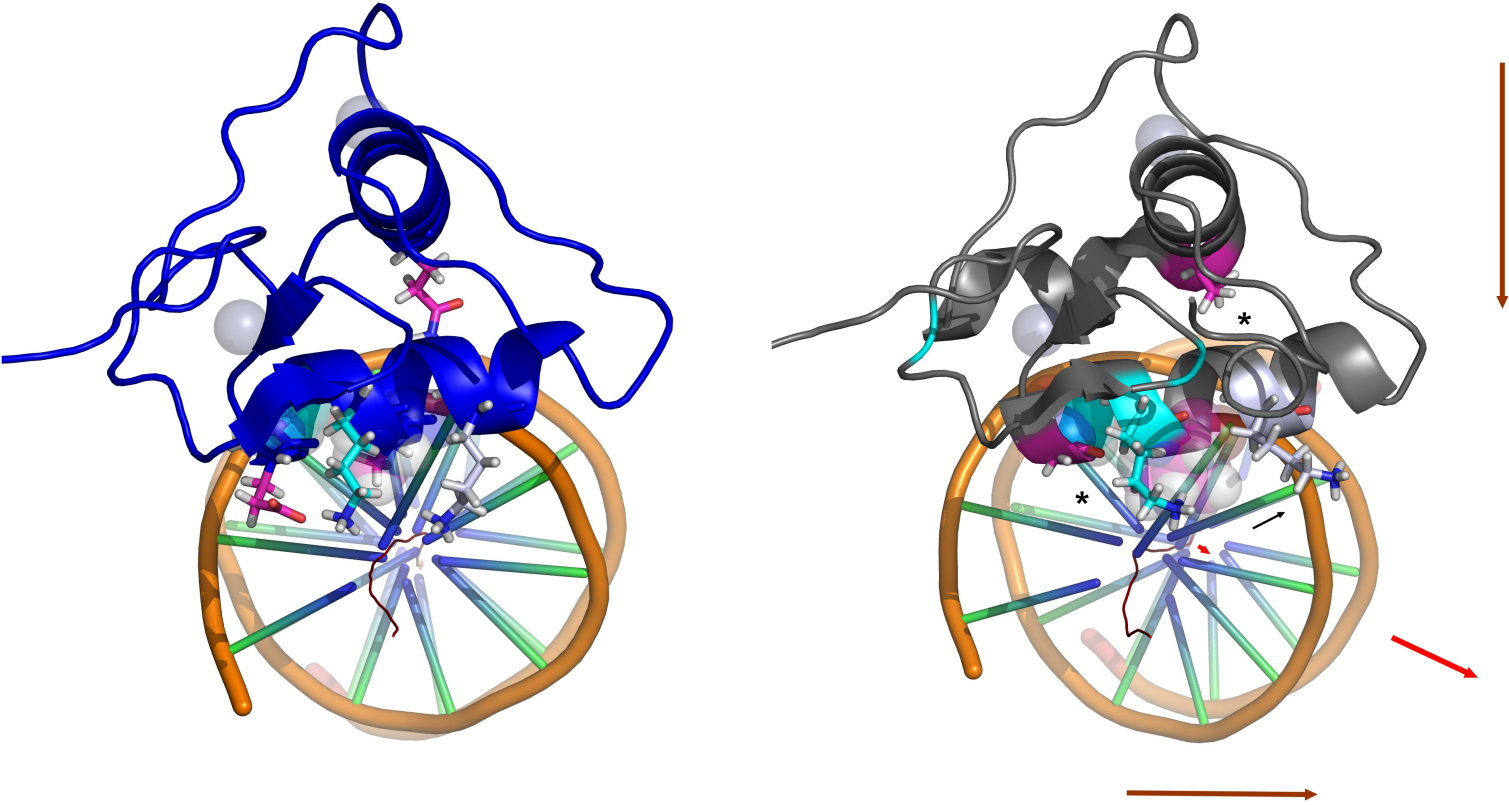
**Divergence on GR residues from set 3, related to the more canonical ER, promotes displacements on the global network of interactions with the half site, concurrent with the variation at DNA curvature**. Brown arrows denote global displacement of the interactions network at GR:GRE (*right)*, these accompanying the variation on the DNA axis curvature (red arrow), compared to ER:ERE (*left*). Asterisks (*right*) signs the shortening of the chains at positions 153 and 188, with significant loss of interactions. The black thin arrow (*right *at the DNA major groove) points to the mutual displacements of the respective side chains from the lysine residues 154 and 160; the first due to the loss of a saline bond to residue 153 (due to the E153G substitution) and the second due to apparent electrostatic repulsion with the first. The atoms of the A157 from ER and of the bulkier V157 from GR (this last packing against nucleotides at the central kink) are depicted as transparent van der Walls spheres.

Finally, the stereochemical dependence of residues 154 and 157 for HRE recognition is evident when comparing structures of complexes with DBDs containing "canonical" P-boxes and structures containing modifications at these positions (Figure 4). Both the structure of the heterodimer of hTRβ:hRXRα on DR4 (Figure 4-A, extracted from PDB:2NLL) and the one of the hTRβ homodimer on TRePal (Figure 4-B, from PDB:3M9E) present DBDs with the archetypical CEGCKG motif bound to archetypical AGGTCA half-sites. It can be noted that the previously cited packing pattern for the G154, G157 and their respective neighborhood adjust themselves considerably well to the half-sites with a DNA axis relatively straight (being such straight axis pattern facilitated, in this case, by the interaction of the hTRβ helical T-box with the minor groove). However, for the homodimer of the estrogen receptor (ER) DBD bound to the ERE (a palindromic disposal of the AGGTCA motif spaced by 3 basis-pairs), the bulkier DBD interface mediated by the two respective D-boxes and the absence of a central T-box contact promotes a bending of the DNA axis toward the major groove at the center of the complex (Figure 4-C). It can be noted that such bent axis causes a relative "slope" of the region of the major groove, at the respective half-sites, where the residue 157 pack against the R161 and this one against the 5' anti-sense thimidine (Figure 4-C). Nevertheless, such "sloped" major groove is very well fitted by the relatively bulkier A157 residue that replaces the G157 at these receptors (presenting the same CEGCKA sequence at the P-box instead of CEGCKG) (Figure 4-C). It is interesting to note that substitutions at A157 for the estrogen receptor has profound effects over the affinity of this receptor to HRE sequences in analogy to what occurs upon the substitutions of G157 for TR, although these residues, themselves, do not contact directly any nucleotide, confirming the stereochemical topological influence mediated by this position.

#### Modifications of the set 3 of the correlation network evidences a role for DBD positional adaptation to DNA axis topology

Correlations between aberrant substitutions at the positions from the sets 2 and 3 of the correlation network and HRE recognition are also particularly evident at the analysis of the structure of the glucocorticoid receptor interacting with its specific HRE (GR:GRE complex, PDB:1GLU) (Figure 4-D). As it is usual for the 3-ketosteroid receptor subclass, GR presents the pattern CGSCKV in its P-box, with substitutions at the three principal positions that significantly modify the stereochemical characteristics of this segment. It is well known that such modifications are accompanied by a change in half-site selectivity, from AGGTCA to AGAACA [31]. A less discussed issue, however, is that 3-ketosteroid receptors present also a subclass conserved substitution at residue 188 of the D-box, from the usual glutamine recovered in this study at the set 3 of the correlation network to a proline residue (Figure 4-D, arrow). Such substitution seems to act in concert with the modified P-box at the specific DBD-HRE fit (*see below*).

**Figure 5:**
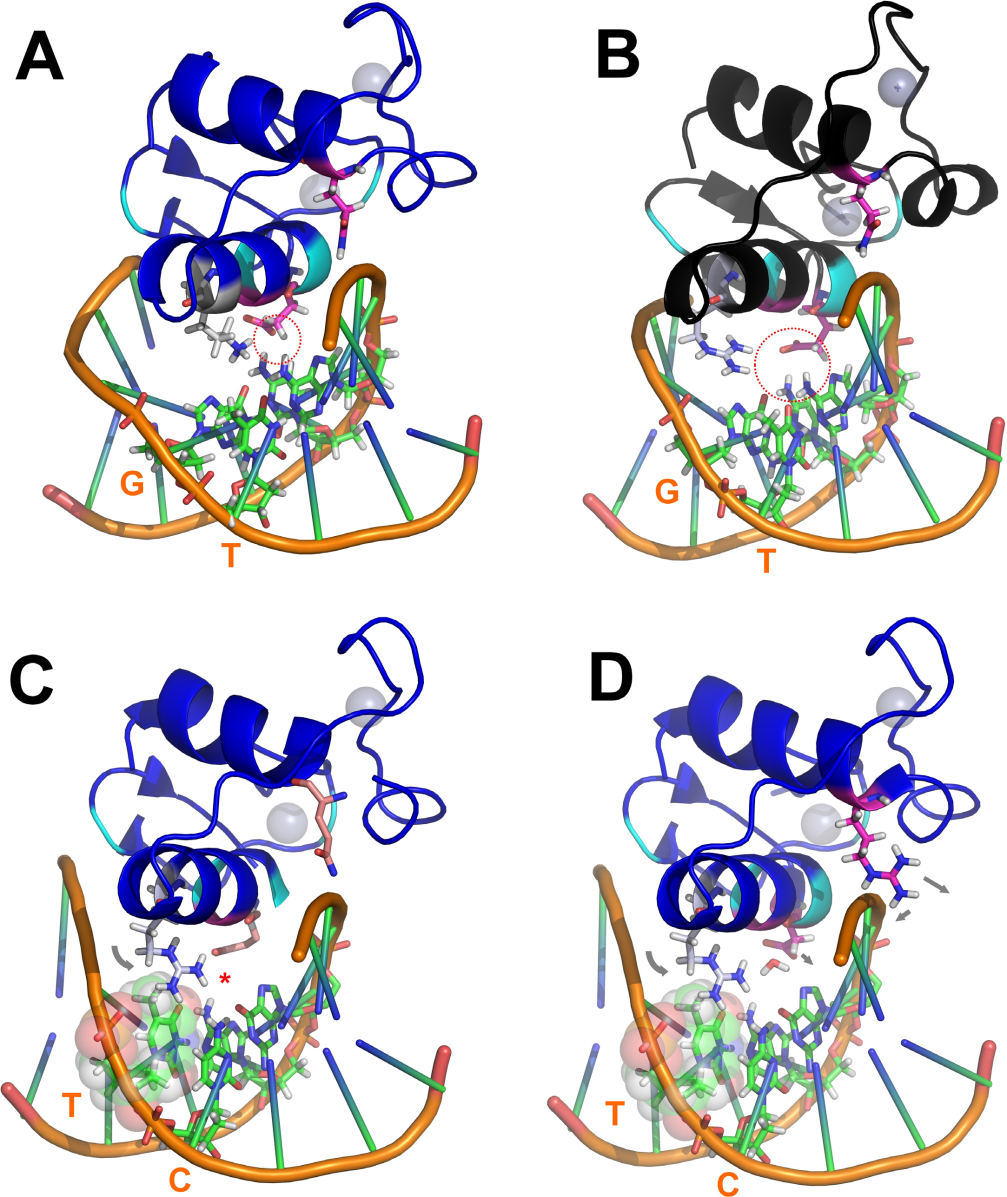
**Simultaneous divergence at position 153 and 188 (from the correlation set 3) for HNF4 promotes a *downstream *displacement of the DBD:DNA interactions network**. *A *and *B *are respectively RXR-DBD (PDB 2NLL) and PPAR-DBD (PDB 3DZY) at canonical half-sites. Red dashed circle show the hydrogen bond distance between E153 and the third antisense cytidine. *C: *model of HNF4 at the specific HRE (AGTCCA) with canonical replacements at the position 153 and 188. The red asterisk shows the loss of hydrogen interaction between E153 and the third anti-sense basis. *D -*The actual HNF4 (PDB 3CBB) presents E153D and Q188R co-substitutions simultaneously with K160R, allowing a global driving of the interaction network at the *downstream *sense and the favorable association with the AGTCCA HRE, HNF4 specific.

A first issue to be considered on an AGAACA HRE sequence is that the placing of the AA central step upstream to a CA step facilitates a local kink, promoting a "depression" of the major groove surface at the region where the recognition helix allocates (Figure 4-D) [32]. The bulky side chain of the V157 residue projects itself over such kink in a more efficient way than the analogous A157 from ER. In this way, the V157 side chain can pack against the C7 methyl group from the anti-sense thimidine of the fourth dinucleotide (van der Walls spheres at the Figure 4-D). The simultaneous bulky substitution of G154 by S154, in turn, contributes by sustaining the H-A mass center in the correct position to allow the placing of V157 over the kink region, which is necessary due to the larger major groove of the AGAACA sequence [32]. The Q188P substitution, in turn, seems to contribute to the stabilization of such system in two ways. Firstly, the reduction of the side chain in contact with the phosphate backbone at this position facilitates the D-box tilting in direction of the dimerization interface with the partner (compare the respective regions at the Figures 4-A to D). Such tilt is necessary for dimerization, both due to the already mentioned deeper and larger major groove for the AGAACA half-site (compared to an usual AGGTCA one) as to the larger spacer between the two half sites for GRE as opposed to ERE (4 bp versus 3 bp, respectively) (Figure 4-C and D). However, a second considerably more interesting movement is allowed by the simultaneous loss of contacts at positions 153 and 188 from set 3 and the gain of contacts at position 157 from the same set in GR (Figure 5). It can be noted that, in order to place the V157 over the "depression" of the major groove, enhancing the protein:DNA contacts and the dimerization between the two DBDs, the web of molecular interactions at the GR-GRE interface should suffer a small displacement, related to the ER:ERE complex. Such displacement can be defined as being along a diagonal crossing the DNA axis and in the direction to the variation on its curvature (Figure 5-B). This movement is facilitated by the simultaneous loss of the saline contacts between K156 and the mutated E153G (K156 is therefore interacting more tightly with the second HRE dinucleotide and pointing in the direction of the displacement), the loss of the hydrogen bonds between E153 and the anti-sense basis of the third basis-pair and, finally, the shortening of the side chain of the residue 188 (with a Q-P exchange) in contact with the phosphate backbone. It can be also noted that, in order to draw back electrostatic repulsion with the displaced K156, the K160 side chain in GR points outside to the major groove, interacting with the phosphate backbone, as opposed to its analogous in ER (and most other NRs) that points inside to the same groove (Figure 5-A and B). Similar modifications are noted at the structure of the androgen receptor (AR) complexed to the 5' half site of the DR3-ARE (PDB 1R4I) and for PR receptor at PRE (PDB 2C7A, not shown).

Therefore, the structural effects of the substitutions at the positions of set 3 in 3-ketosteroid receptor DBDs suggest an evolutionary pressure related to the optimization of the correct placing of the domain related to the modified DNA principal axis of its HRE, in order to maintain satisfactory interactions with the half-site and between monomers. It is interesting to note that such divergent pattern of the set 3 and set 2 (considering the position 154) for keto-steroid receptors does not appear at the correlation scheme found in Figure 1, apparently as a direct effect of the evolutionary history of 3-ketosteroid receptors. They are part of the NR3 subfamily, which is exclusive to vertebrates, and the current diversity of this subfamily (two estrogen receptor isoforms, an androgen receptor, a mineralocorticoid receptor, a glucocorticoid receptor and a progesterone receptor) was the result of duplication and diversification from an ancestral estrogen receptor [31]. Being much more recent than the receptors containing a CEGCKG or similar motif, the very high similarity for the DNA binding domains of 3-ketosteroid receptors means that, in order to produce a well sampled final alignment, the procedures described in *Materials and methods *remove most of those receptors from the DBD alignment, resulting in only five sequences with a CGSCKV motif.

**Figure 6:**
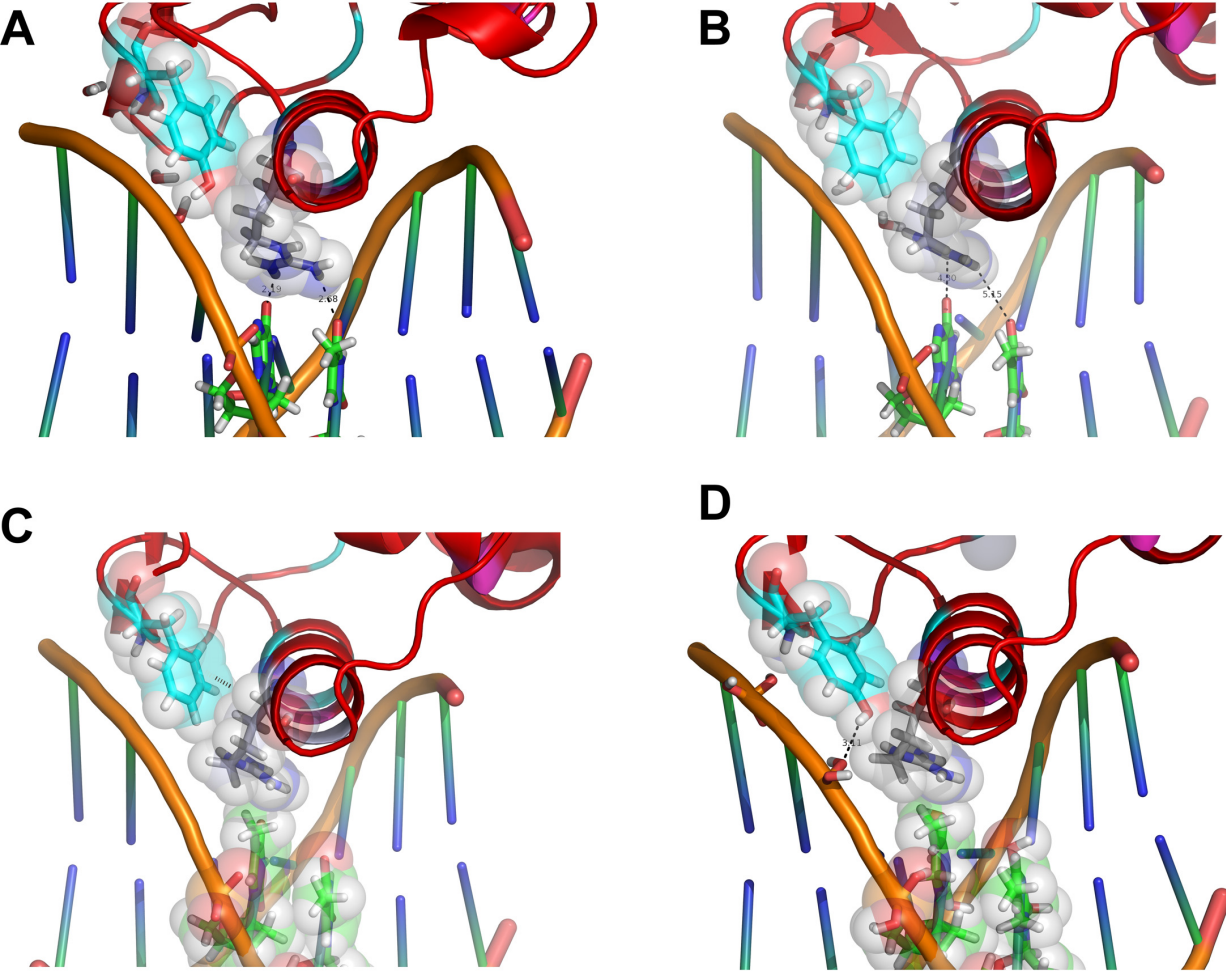
**Role of the T147F and K160R co-substitution at the recognition of NNTTCA half sites**. *A: *hTRβ on TRE (PDB 2NLL) showing double hydrogen bonding between R160 and the central basis step. *B: *VDR on canonical AGGTCA half site (PDB 1KB4), showing packing of R160 against the T147F substituted residue, which, in turn, move the R160 away from the central basis step and distort the above cited hydrogen bonds. *C: *VDR on AGTTCA half site (PDB:1KB2). R160 is over-packed between the C7 atom from the thimidine at the third basis-pair and the F147 side chain. *D*: model of VDR with a F147T substitution showing distorted interaction of the T147 with the backbone due to stereochemical hindrance.

Another evidence from literature of the concerted role of the set 3 residues on global orientation of the DBD:DNA web of interactions can be found at the structure of the less divergent HNF4 DBD:HRE complex (PDB:3CBB) [32]. The HNF4 nuclear receptor is able to bind, with strong affinity, to an alternative HNF4 specific half-site with the 5'-AGTCCA-3' sequence [32,33]. Mutational studies [33] have demonstrated that such divergent selectivity is well correlated with the presence of two non-usual modifications at the HNF4 DBD: an E153D substitution (at the 1^sth ^P-box position, corresponding also to the first position at the set 3 recovered on our correlation network) and a not so unusual K161R substitution (present in another NRs such as TR, VDR and PPAR, which are unable to bind to this kind of half-site with significant affinity). In the cited study, the greatest effect on HNF4-specific binding was mediated by the E153D substitution, although the R161 presence seems to have an optimizing role. It was also observed that the D153-R160 charged pair, in humans, is only found on HNF4 DBDs. However, a non discussed issue is an even more ubiquitous substitution, at position 188 of the set 3 for HNF4 DBDs: a Q188R substitution, that is frequently found even on *C. elegans*, and that when absent in this organism is substituted almost exclusively by another basic K188 residue, preserving a considerable part of its physical-chemistry (see discussion at the next topic). Comparing the set of interactions mediated by these three positions (153, 160 and 188) for canonical and HNF4's DBD:HRE interactions we can suggest an integrated role for these three residues at the HNF4 positioning on it divergent half-site (Figure 6). Figure 6-A-B, depicts, respectively, the structures of the DBD:(5'-AGGTCA-3') complexes for hRXRα (PDB:2NLL) and hPPARγ (PDB:3DZY), in both cases highlighting the hydrogen bonding distance between the E153 residue (from set 3) and the anti-sense basis for the half-site third pair (dashed red circle). Also visible is the stacking, in hydrogen bonding position, of the Q188 residue of these two DBDs over the respective DNA backbones. Finally, the basic residue at the 160 position (a lysine for RXR and an arginine for PPAR) are both positioned in order to make moderate interactions with the hydrogen bond acceptors on the central basis step (GT), as usual for canonical DBD:HRE contacts at this position. On the other hand, on a model based on the structure of the HNF4-DBD complexed with its specific HRE (AGTCCA) (PDB 3CBB), but with the residues 153 and 188 manually replaced by their respective E and Q canonical constituents (Figure 6-C), it can be noted the loss of an hydrogen bonding favorable position between E153 and the anti-sense third basis, due to the cytidine-adenosine substitution at this site (red asterisk). It can also be noted that R160 packs against the sense thymidine, directing the Arg shift basis in a *downstream *sense (gray curved arrow), drawing back interactions with the third base hydrogen acceptor (being the N6 atom at the fourth base the single one hydrogen acceptor available). Such pattern of missing hydrogen bonds is concurrent with the low affinity of DBDs containing the canonical E153 residue with AGTCCA half-sites. For a real HNF4-DBD, however (Figure 6-D), the E153D substitution mediates the shortening of the side chain of this acid residue, which in turn facilitates water molecules insertion that can be accommodated between it and DNA, enabling water mediated hydrogen bonds with the fourth anti-sense base (gray arrows at Figure 6-D). This directs the interactions between the D153 residue and DNA also in a *downstream *sense (in a congruent way with which occurs for the R160 set of interactions). Still, an interesting issue is that the less discussed Q188R substitution at this receptor makes possible that the side chain of this residue can extend itself over the minor groove, in the same *downstream *direction that the other two substitutions at the recognition helix. Beyond it, the longer, positive charged and bidentate chain of the Arg residue promotes stronger interactions to the backbone phosphates, apparently compensating the relatively labile interactions of the D153 residue with the DNA (gray arrows at Figure 6-D).

Although the influence of substitutions at the HNF4 R188 or GR P188, or still any other DBD 188 position over the HRE specificity has still not been systematically checked, such structural analysis, taken all together, corroborates that residue substitutions at the set 3 of the correlation network could act in a concerted way to adapt the DBD positioning to aberrant DNA contexts.

#### An alternative residue subset including a divergent P-box for Caenorhabditis elegans, and insights about the role of the T147F substitution

What is particularly striking is the appearance of residue set 1 (F147, R153, A154, A156 and A157) in the analysis. The resulting P-box motif (CRACAA) appears exclusively on *C. elegans *and *C. briggsae*, even though the final alignment is populated by other sequences from class *Chromadorea *(*Brugia malayi*, for example, is present with fourteen sequences). A search using all available sequences shows that this is true both for CRACAA and the physical-chemical similar motif CKACAA, with 178 and 18 available sequences, all of them from the *Caenorhabditis *genus. These results show that even though the explosive expansion of the nuclear receptor family in nematodes was accompanied by enough diversification in order to keep a statistically significant sequence number after the cutoffs described in *Materials and methods *(which were strict enough to reduce 3-ketosteroid receptors to a minimum), it is still noticeable that many of them kept conserved a specific *P-box *motif that is only found in this clade - which suggests that, analogous to what happens at the 3-ketosteroid receptors subclass, this group of nematode nuclear receptor may have evolved a different binding mode for DNA response elements.

**Figure 7:**
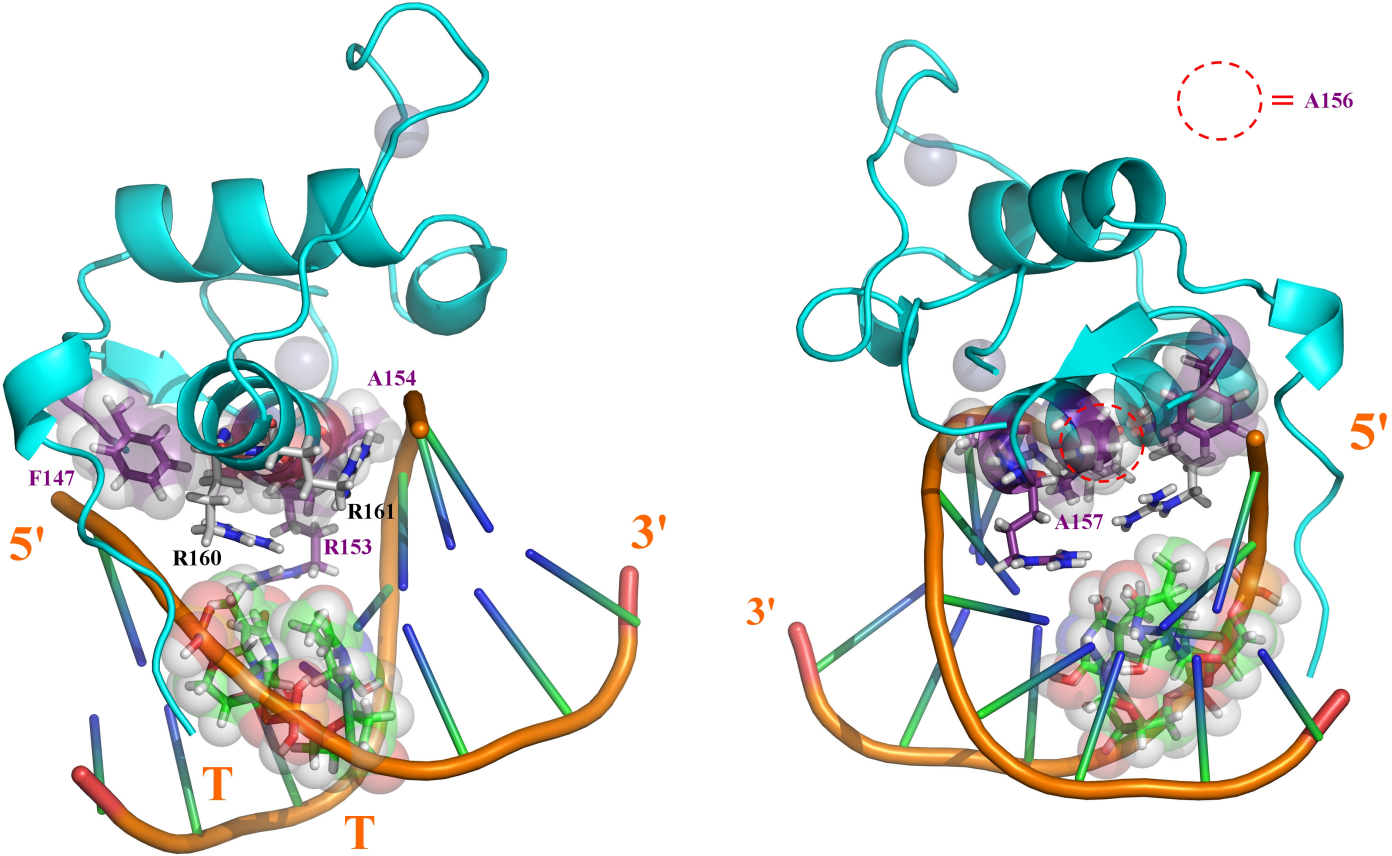
**Divergent pattern of the set 1 in a model for a representative *C . elegans *DBD, as viewed at two different angles at a complex with a hypothetical 5'-NNTTCA-3' half site**. It can be noted the over-packing of the R160 residue between the thimidine at the third dinucleotide and the F147, in an analogous way that the VDR-DBD at a similar half site (Figure 6-C). The DBD modeled was NHR28. The half-site used was from the 3' side of the HNF4:(MODY related promoter) complex (PDB 3CBB). Residues from the set 1 of the correlation network are depicted with the carbons colored in purple (and labeled at this same color) while the two more conserved basic positions R160 and R161 (that do not appear at the correlation set) are depicted with carbons in white and labeled in black. For clarity, in the picture at the right residue A156 is identified with a dashed red circle.

Figure 1 shows that not only the P-box motif has been conserved in this NR subset, but the presence of this motif also increases the chances of finding a phenylalanine in position 147 (while other receptors would have T147, from set 2, in that position). The only "canonical" NR-DBD structure presenting such modification is the one of vitamin D receptor (VDR). Another particularity of VDR that is also present at the divergent NRs of *C. elegans *is the substitution of the typical K160 by a less typical R160 (similarly to what happens for TR, PPAR and HNF4). Actually, in *C. elegans *there is an inversion of the frequencies for the residues at position 160 for DBDs that have a tyrosine at position 147; these presenting 100 % of Lys at position 160 (as expected for non divergent DBDs), as opposed to 99.4 % Arg and 0.3 Lys for the subset of *C. elegans *DBDs containing the set 1 of residues from the correlation network (which, in turn, presents a Phe at position 147). In analogy to what occurs for the 3-ketoseteroid divergent P-box, the rise on the K160R frequency associated with set 1 is not evident on the correlation network at Figure 1 due to low sampling for this class. An interesting issue is that the presence of an arginine at position 160 in NRs is, in turn, well correlated with the possibility of alternative binding, generally with lower affinity, to PuGTTCA half sites (Pu being a purine nucleoside), as is observed, for instance, for TR [29,30] and for HNF4 [32,33]. VDR, however, still presents a higher affinity for PuGTTCA when compared to PuGGTCA half-sites [37]. An analysis of the respective structures of TR and VDR on AGGTCA half sites (respectively, PDB 2NLL and PDB 1KB4, Figure 7-A-B); VDR on the osteopontin promoter containing GGTTCA half sites (PDB 1KB2, Figure 7-C) and a model of VDR on GGTTCA in which the F147 is manually substituted by the canonical Tyr (Figure 7-D) suggests that the simultaneous presence of the R160 and F147 in VDR takes a crucial participation on such half site discrimination, which was not originally taken into account in [34]). At Figure 7-A, it can be noted that the R160 residue of TR-DBD makes simultaneous charged hydrogen bonds with the hydrogen acceptors on the central basis step, while the phenolic T147 is packed between the H-A and the DNA backbone, with its hydroxyl group making favorable hydrogen bonds with the DNA backbone. Packing of the T147 is sufficiently soft to still allow that structural water molecules (in sticks representation at the Figure 7-A and B) surround the polarized Tyr residue. For the VDR-DBD, however, (Figure 7-B) the T147 is substituted by a hydrophobic phenylalanine residue, that in order to optimize its packing and limit accessibility of surrounding waters, recruits the aliphatic portion of the R160 chain from VDR. Such packing of the aliphatic portion of R160 against F147, in turn, departs and distorts the interaction of R160 with the hydrogen acceptors on the central step, drawing back such hydrogen interaction. It is expected that such loss of two charged hydrogen bonds contributes significantly for the lower affinity of VDR to canonical half sites compared to TR and analogs. At the interaction of VDR with the modified PuGTTCA, however, the VDR's R160 side chain packs against the C7 methyl group of the thimidine at the third pair (this one, in turn, stacking against the subsequent thimidine at the fourth pair), sustaining the same R160 in the correct position to pack favorably against the aromatic F147 and the recognition helix backbone (Figure 7-C). Such over-packed system seems to be very energetically favorable, considering the hydrophobic interactions involved. Finally, when the canonical F147T substitution is introduced at the GGTTCA interaction, the steric hindrance mediated by the hydroxyl group promotes distortion at the stacking between the two residues (Figure 7-D). Hence, the minimally favorable conformation still simultaneously preserving the T147 interaction with DNA and the H-A comprises the polar hydroxyl group stacked against the non-polar R160 aliphatic chain, and is in a relative distance and angle from the nearest backbone groups that turn hydrogen bonds impracticable. This is concurrent with the apparent lower affinity of NRs having a Tyr at site 147 (from set 2) with PuGTTCA than for PuGGTCA half sites, in opposition to what seems to occur (considering VDR) for the ones that present an F147 (from set 1). Beyond this, the effects of such T147F substitution seems to be enhanced by R160, also very usual for the *C. elegans *NRs presenting the residue pattern from set 1 recovered by our correlation network. In this sense, it is a reasonable assumption that such divergent nematode DBDs evolved parallel to the "canonical" ones, in order to recognize preferentially 5'-NNTTCA-3' sequences, the nature of the 5'-NN nucleotides depending from the other modifications and, probably, from NR specific context (C-terminal extension, dimerization, etc.). The preferential recognition of 5'-NNTTCA would be not a surprisingly factor, since that it is proposed that the divergent *C. elegans *NRs evolved from a modified lineage of HNF4 [4,5], the HNF4 itself being able to associate (with lower affinity) to AGTTCA half sites (in fact, some important HNF4 activated promoters in humans, involved on the pathology knew as *maturity onset diabetes of the young (MODY)*, contain such modified half-site) [32,33].

#### The aberrant E153R substitution at set 1: implications for divergences on the first basis step and half site topology

The more intriguing substitution at the first set of the correlation network however is, without a doubt, the E153R one. Such substitution changes, simultaneous and drastically, the nature of the charge (from negative to positive), the pattern of interaction at hydrogen bonds (from acceptor to donor) and the extension of the side chain (from a four sections chain - two aliphatic and a carbonyl carbons plus the terminal acid oxigens - to a six sections one - three aliphatic carbons plus a nitrogen and a carbon from the basis plus the two terminal nitrogens); beyond to promote an enlargement of the terminal bidentate group (due to the addition of two protons at each terminal nitrogen on the basis, compared to the non-protonated oxygens at the acid group). In face of such accentuated physical-chemical and stereochemical changes, it is equally expected drastic changes at the topology and physical-chemical ambient of the complementary DNA sequence, implying on additional modifications on half site specificity. The co-substitution of the Gly residues at positions 154 and 157 by Ala is concurrent with the necessity of global topological changes on the major groove (as previously discussed, see Figure 3) for accommodation of the substitutions at set 1. In particular, R153 presents both a correlation with an Ala at position 157 as well as an anti-correlation with a Gly at the same position (Figure 1). Due the localization of the 157 residue near the center of the recognition helix (projecting itself near the center of the half-site major groove) (Figures 2, 3 and 7-A), it is expected that the side chain at this position presents more profound correlations with the global DNA topology (see, for instance, the global topological modifications that are accompanied by a G157A substitution on the ER:ERE complex, in Figure 3-C). As already discussed, the E153R substitution is anti-correlated with the presence of the K156 residue (Figure 1), which is justified by the physical proximity of the two residues, establishing an important saline bond when a Glu and a Lys are present at the respective positions (Figures 2 and 4). So the presence of the positively charged and long chain R153 on set 1 is accompanied by a K156A substitution that removes the positive charge and shortens the side chain at the site 156. A second inference that can be taken about such co-substitution is that the R153:DNA contacts should compensate the loss of the K156:DNA ones, *i.e*. R153 must interact with the first basis step of the half site (see Figure 2). The G154A bulky substitution at the opposite side of the half-site could, in turn, contribute to "push" the DBD mass center in the *upstream *direction (Figure 7) in order to facilitate the interaction of the R153 with these *upstream *basis, and even the packing of the R160, on the other H-A extremity, against the F147 residue (Figure 7), in an analogous way that the G154S substitution on 3-keto-steroid receptors seems to facilitate the positioning of the substituted G157V over the kink region on Figure 2-D. It is also interesting to note that the set of alanine residues at positions 154, 156 and 157, and the phenylalanine at position 147 form a hydrophobic "belt" between the recognition helix and the backbone wall, which could be helpful to adjust the H-A at the surface of the half site, while the three "deeper" arginine residues at the helix extremities (R153, R160 and R161) mediate the specific protein-basis contacts in a symmetric way (Figure 7).

Hence, the half site specific adaptations inferred for the E153R substitutions (*i.e*., global topological alterations and possible modifications on the 5'-NN first basis step) are, in principle, permissive for the adaptation individually inferred due the simultaneous T147F and K160R substitutions (*i.e*., a GT-TT dinucleotide step substitution at the center of the half site). Although the sequence based prediction of protein:DNA specificity is still a hard, and in a certain way risky, task (due the common superposition of long range topological terms over the local features[35,36]), a goal for future studies would be to test the affinity of representative *C. elegans *DBDs for the 16 possibilities of the 5'-NNTTCA-3' half site and for more canonical ones (AGGTCA, AGAACA and AGTCCA), as well as the search for such sequences on *C. elegans *promoters (and from related organisms).

#### An apparent role for the 140 and 188 residues (and for sets 2 and 3 as a whole) in counter-balanced interactions related to the DNA-axis along the evolution

Finally, the statistics of residue prevalence at positions 140 and 188 (that do not appear at the set 1 of the correlation network) in the DBDs whose sequences fall at this same set, reveals interesting aspects of an apparent co-evolution of these two positions and from them and the rest of the correlation network. The great majority of the PFAM sequences used in our correlation analysis present the residues corresponding to sets 2 and 3, hence, with a glutamine at position 188 and an aspartate at position 140. As previously discussed and considering just DBDs that do not share the divergent set 1, the position 188 present some less frequent modifications, or as part of an alternative group (the case for the P188 of the 3-keto-steroid receptors) or as an individual discrepancy (as for the R188 of HNF4). Surprisingly, D140, which does not interact with DNA and that is repulsive for the backbone phosphates, is significantly more conserved between the DBDs that do not share the set 1. For the *C. elegans *DBDs presenting the set 1 however, while the position 188 present almost exclusively basic residues (46.6 % of Arg and 42.9 % of Lys), the position 140 has an accentuate variance, allowing almost all of the 20 aminoacids except prolines and tryptophans. The more prevalent residues at this position on *C. elegans *are Glutamine (25.7 %), Serine (12.5 %) and Glutamate (11.0 %), ranging, so, significantly, in chain size, charge and hydrogen bonding pattern even between the three more representative residues.

**Figure 8:**
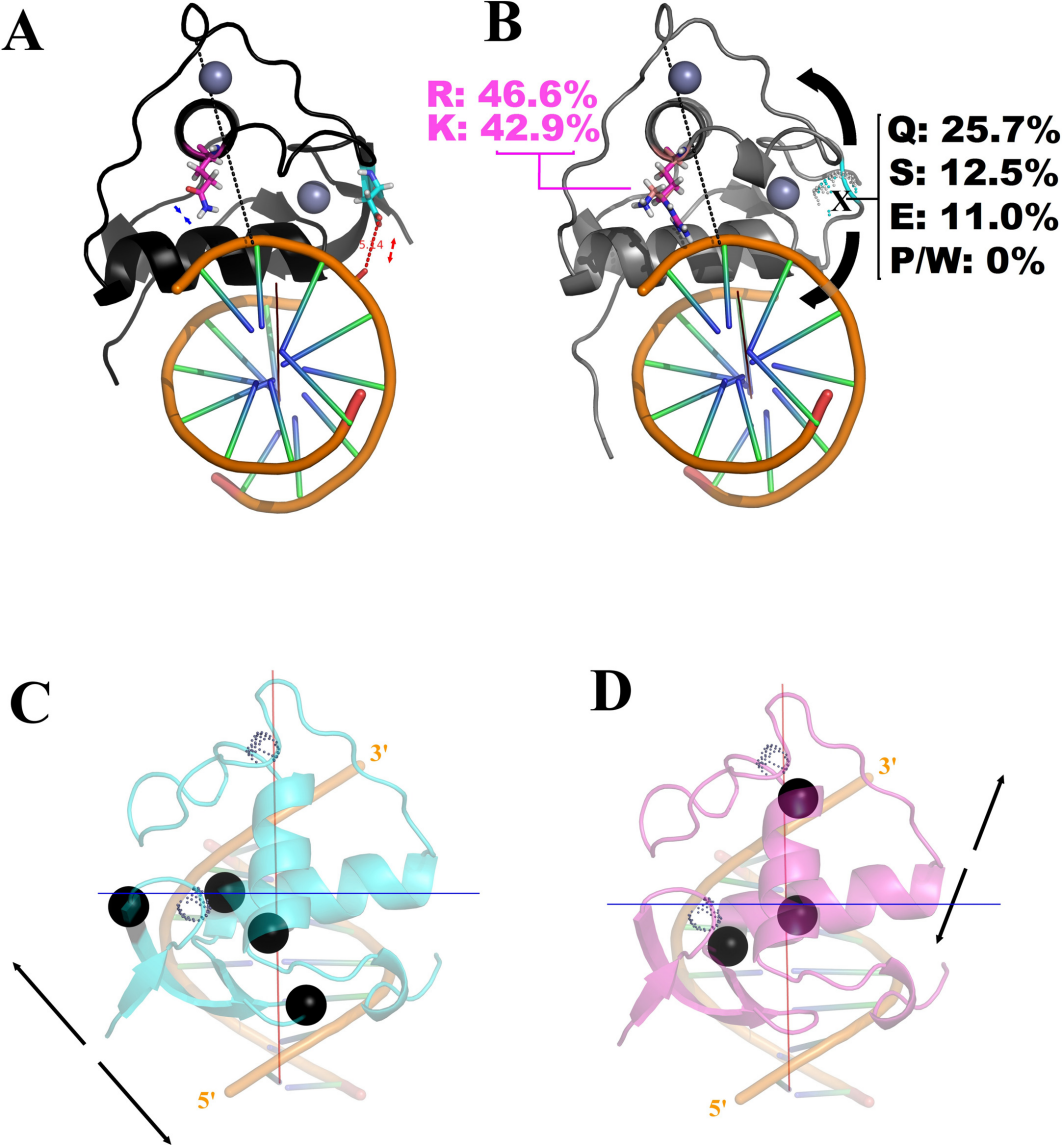
**Trans-axial disposition of the interaction-network orchestrated by the sets 2 and 3**. In *A *and *B*, the nature of the trans-axially positioned 140 and 188 residues are shown, respectively, for canonical and divergent DBDs. In *A*, the distance from the D140 acid oxygen atoms to the nearest backbone phosphate (repulsive to that residue) is assigned (5.4 Å). In *C *and *D*, the residues from, respectively, sets 2 and 3 (*black spheres for the C_α _atoms*) regulate interactions with the major groove along distinct diagonals (*double arrows*) passing through the principal axes from the complex. The DNA major axis (red) was calculated as described in *Methods *and the blue perpendicular axis was traced in order to pass through the H-A geometric center. Dotted spheres represent the Zn^2+ ^ions. Canonical B-form fragments of DNA were used for all the respective models (*see text for details*).

Figures 8-A and B depict, respectively, model-structures of complexes with major groove's half sites (considering an archetypical B-form DNA) for a typical DBD with sets 2 and 3 and for a *C. elegans *DBD with the set 1. The positions of residues 140 and 188 are highlighted in both structures. It can be noted that these two residues present themselves in opposite sides considering both the DNA principal axis, as an imaginary line passing by the D-box mass center and the nearest phosphate at the DNA's backbone wall (this line, in turn, dividing the DBD volume nearly by the half). For typical DBDs, while the interaction at the site 188 is attractive, the one at the site 140 is moderately repulsive (the negative acid residue being at an average distance of 5-7 Å related to the also negative phosphate in the backbone). In fact, the D140 residue is surrounded by basic residues that interpose between it and the DNA backbone, hence masking its repulsive contribution. In this way, the set of interactions perpendicular to the DNA and the DBD principal axis are more favorable at the Q188 side. For the divergent DBDs from *C. elegans*, however, the ranging on the size and the coordination number of the basic residue at the 188 site (a monodentate lysine or a bidentate arginine) plus the high variance on the physical-chemistry at the 140 site promotes a significant variation on the way in which the interactions at each side of the axes are counterbalanced for different DBDs. It is expected that such pattern allows small variations on the angle in which the recognition helix fits the major groove for different DBDs (curved arrows at the Figure 8-B), which in turn can be used at the adaptation for different dimerization partners and/or different half-sites (for instance, allowing that the large chain of the R153 adjust itself for a different 5' basis step).

Another indication that the counter-balancing of interactions along the DNA and the DBD principal axis seems to be a driving force along the evolution is the pattern at which the residues from set 2 and 3 distribute themselves along the major groove interaction (Figure 8C and D). Both sets of residues are located at different diagonals crossing the DNA principal axis (red vertical axis at Figures 8-C and D); in order to stay, mostly, at different sides considering also an axis perpendicular to the first and passing through the H-A center (therefore dividing the major groove approximately by the half, blue horizontal axis at Figures 8-C and D). In this sense, each set controls interaction networks that, in principle, modulate the positioning of the DBD at each one of these respective diagonals (double arrow at Figures 8-C and D) and that, taken all together, can regulate small translation and rotation movements for the DBD around the entire major groove's inclined plane.

Taken all together, the correlation analysis for the DBDs indicates that evolutionary correlations for this domain are implicated on global fitting at the DNA topology, superposing such long range effects over the local interactions.

## Conclusions

The correlation analysis of nuclear receptors presented here made possible the discussion of many residue-specific features in this protein family that could not be easily achieved from methods using just positional conservation. While LBDs were previously analyzed by such methods, the residue-specific approach revealed a much detailed picture for the relations between the different functional surfaces and class-specific residues. For DBDs, there is a strong clade-specific element in the correlation analysis, which, when analyzed in the light of the many structures of DNA bound DBDs, opens interesting possibilities for the understanding of hormone response elements specificity. We expect that such effort will be helpful in understanding the functional evolution of NRs, as well as to broaden the knowledge on the mechanisms of specific protein:DNA co-adaptation, an also on the molecular evolution of nuclear receptors.

## Methods

Multiple sequence alignments of DNA binding domains (DBDs) were downloaded from the PFAM database (PFAM Code: PF00105). To avoid the presence of fragments or too divergent sequences, a minimum alignment coverage of 80% and minimum identity of 15% were imposed to all sequences, using human RXR-α as reference sequence. In order to reduce phylogenetic bias, an identity cutoff of 80% was applied. These procedures reduced the alignment size from 3702 to 508 sequences. Pairwise correlation scores were calculated for all residue+position pairs which were present on at least 25% of the sequences (minimum sub-alignment size was calculated as described in [37]). The correlation score was measured using -log(P) for correlation and log(P) for anti-correlation, where P is the p-value corresponding to the binomial probability of observing the corresponding frequency shift [15]. Twelve residue-position pairs can be arranged in a network where a connection is added where a significant correlation or anticorrelation (minimum score = 10, Δf = 0.30, see [15] for details) is present. The resulting network is seen in Figure 1.

Ligand binding domain sequences were obtained from the PFAM database (PFAM code: PF00104), and given their higher variability when compared to DBDs, were filtered using 50% alignment coverage, but the same values for minimum (15%) and maximum (80%) identity. The final alignment consisted of 1042 sequences, with correlations being calculated with a cutoff score of 10, minimum alignment size of 20% and Δf = 0.30. The resulting network was considerably larger (49 nodes, 61 connections) than the one found for DBDs, requiring the use of heuristics for network decomposition by community detection [16-18]. Nineteen residue-position pairs were successfully grouped into four communities, while the remaining thirty residues remained isolated (due to presenting anti-correlation connections only). Software used for conservation and correlation calculations is available to academics under request.

Protein figures were prepared using PyMol (Delano Scientific), which was also used for manual amino acid substitutions in protein models. The DNA major axis was calculated using *Curves *[38]. Canonical B-form models for DNA half sites (used at Figure 8) were built using Coot[39] and the DBD was manually aligned in the complex form's position at such models using Pymol (http://www.pymol.org).

## Supporting information

The final alignments for DBDs and LBDs are included as supporting files in PFAM format. A list of the proteins with available three dimensional structures is also provided.

## Competing interests

The authors declare no competing financial, professional or personal interests which may have influenced any content of this manuscript.

## Authors' contributions

MQLA performed the correlation analysis from multiple sequence alignments, drafted and analyzed the results. LHFL performed structural analysis and wrote the sections related to DNA binding domain structures. LB designed and supervised the study, and wrote the remaining sections. All authors read and approved the final manuscript.

## Declarations

Publication for this article has been funded by the Brazilian funding agency *Conselho de Aperfeiçoamento de Pessoal de Ensino Superior *(Capes).

This article has been published as part of *BMC Genomics *Volume 14 Supplement 6, 2013: Proceedings of the International Conference of the Brazilian Association for Bioinformatics and Computational Biology (X-meeting 2012). The full contents of the supplement are available online at http://www.biomedcentral.com/bmcgenomics/supplements/14/S6.
